# Harm-Reduction for Substance Misuse in Young People: A Service Evaluation of Southampton's Drug and Alcohol Support Hub (DASH)

**DOI:** 10.1192/bjo.2024.97

**Published:** 2024-08-01

**Authors:** Camilla Walker, Charlotte Primavesi, Pelumi Popoola, Emily Walmsley

**Affiliations:** 1 Camden and Islington, London, United Kingdom; 2 Sailsbury NHSFT, Sailsbury, United Kingdom; 3 University Hospital Southampton, Southampton, United Kingdom; 4 University of Southampton, Southampton, United Kingdom

## Abstract

**Aims:**

This project aims to evaluate Southampton's Drugs and Alcohol Support Hub Service (DASH) for young people (YP) provided by the charity, No Limits. It aims to produce insights and recommendations for No Limits to improve their service for YPs and positively influence local commissioning and governmental bodies. This project was part of the Wessex Public Health Fellowship for Junior Doctors, which aims to provide experience of working in public health and teach relevant research skills.

**Methods:**

An adapted-Donabedian framework was implemented and a review of the literature informed a ‘harm-reduction’ lens for analysis. Mixed methods were used: Quantitative analysis reviewed data from 50 (anonymised) YPs. All data were routinely collected by No Limit's staff as Young People Outcome Records (YPORs) and Client Information Reviews (CIRs), as well as outcome measures collected quarterly for the National Drug Treatment Monitoring Service (NDTMS). Qualitative methods included a thematic analysis of five semi-structured interviews with service providers.

**Results:**

Cannabis and alcohol were the most commonly reported problem drugs for YP (48% and 36%, respectively). In terms of smoking per weekdays, 67% of YPs were using cannabis for the same number of days and 15% had decreased smoking days. For smoking in grams, 26% were smoking the same amount of cannabis compared with 41% smoking less. For alcohol, 41% consumed fewer units and 44% had increased alcohol-free days. Importantly, 63% of YPs reported increased quality of life and 59% increased happiness.

Thematic analysis generated seven themes: harm reduction, mental health, relationships and trust, inter-agency working, YP-led care, individual outcomes and differences between reported outcomes and care provided. Harm reduction for most meant helping the YP build healthier relationships with drugs vis-à-vis enforcing abstinence. Trust was necessary for service providers to support YP reach their goals and YP-led, individualised goals benefitted most. Next, service providers often supported YP with mental health and sometimes this created challenges beyond their professional capabilities, thus emphasising the importance of collaborative inter-agency working. Lastly, providers were frustrated with required NDTMS outcome measures given they failed to capture service benefits.

**Conclusion:**

DASH service's ‘harm reduction’ approach to supporting YP with substance misuse is in-line with evidence-based best practice guidance. However, reported NDTMS outcomes remain driven by an abstinence-informed agenda. This policy is grounded in governmental policies that do not consider the nuance of substance misuse disorders and are reflective of Nancy Reagan's 1980s ‘Just say No’ campaign. To prioritise the health and mental health of young people, government must reframe their policy on substance misuse.

